# Cefepime 2.0: Upgrading β-Lactamase Inhibitor Use

**DOI:** 10.3390/microorganisms14091875

**Published:** 2026-08-24

**Authors:** Francesco Giuseppe De Rosa, Tommaso Lupia, Valentina Fornari, Davide Vita, Marco Casarotto, Silvia Corcione, Alessandra Oliva

**Affiliations:** 1 Department of Medical Sciences, Infectious Diseases, University of Turin, 10126 Turin, Italy; 2 Infectious Diseases, University Hospital Città Della Salute e Della Scienza di Torino, 10126 Turin, Italy; 3 Division of Medicine, Tufts University School of Medicine, Boston, MA 02111, USA; 4 Department of Public Health and Infectious Diseases, Sapienza University of Rome, 00185 Roma, Italy

**Keywords:** antimicrobial stewardship, cefepime, antimicrobial resistance

## Abstract

Antimicrobial resistance (AMR) in Gram-negative pathogens represents a critical global health challenge. Combining cefepime with novel β-lactamase inhibitors (enmetazobactam, taniborbactam, zidebactam, and nacubactam) revitalizes the role of this fourth-generation cephalosporin in contemporary therapy. We present a narrative review according to the Scale for the Assessment of Narrative Review Articles (SANRA) criteria, summarizing the existing literature regarding new cefepime combinations with β-lactamase inhibitors. Based on 68 identified studies, data were structured by molecule. Each section explores in vitro activity, preclinical in vivo data, PK/PD parameters, clinical evidence, and dosage. Findings demonstrate that these inhibitors successfully restore cefepime’s efficacy against diverse resistance mechanisms, notably ESBLs, AmpC, KPC, OXA-48, and metallo-β-lactamases. “Cefepime 2.0” therapies expand the therapeutic armamentarium against severe multidrug-resistant infections, offering vital carbapenem-sparing strategies. Because they possess distinct microbiological spectra, these agents serve complementary rather than interchangeable roles. Their clinical success demands targeted integration into antimicrobial stewardship programs to optimize efficacy and safely reduce carbapenem dependence.

## 1. Introduction

The latest updates from global health organizations underscore the escalating and devastating trajectory of antimicrobial resistance (AMR), positioning it as a preeminent global health crisis [1]. According to the 2025 World Health Organization (WHO) Global Antimicrobial Resistance and Use Surveillance System (GLASS) report, approximately one in six laboratory-confirmed bacterial infections worldwide is now resistant to standard antibiotic treatments, a ratio that worsens to one in three for urinary tract infections [2]. This crisis is disproportionately driven by Gram-negative pathogens; for instance, resistance to third-generation cephalosporins has surpassed 44% in *Escherichia coli* and 55% in *Klebsiella pneumoniae* bloodstream infections globally. Furthermore, epidemiological forecasts project that if current trends persist without significant intervention, AMR could directly account for up to 39 million deaths between 2025 and 2050 [1,2]. The rapid dissemination of carbapenem resistance, coupled with an average annual resistance increase of 5% to 15% across critical bug–drug combinations, emphasizes the urgent clinical necessity for novel therapeutic strategies to mitigate this syndemic of resistance and preserve the efficacy of vital antimicrobial agents [1,2].

Cefepime (FEP), a semi-synthetic fourth-generation cephalosporin with broad-spectrum activity against both Gram-positive and Gram-negative bacteria, was first approved in Europe in 1993 for pneumonia, complicated urinary tract infections (cUTIs), skin and soft-tissue infections (SSTIs), complicated intra-abdominal infections (cIAIs), and neutropenic fever [3,4]. Similarly to other β- lactams, FEP inhibits the synthesis of the bacterial cell wall by attachment to penicillin-binding proteins (PBPs), thus impeding the transpeptidation of peptidoglycan. As in other antimicrobials, monotherapy with FEP has disadvantages: selection of Amp-C, although the drug is intrinsically more resistant than other beta-lactams; variable activity against ESBL-producing bacteria; hydrolysis by carbapenemases except for OXA-48, although frequently co-expressed with ESBL enzymes; and variable activity against *P. aeruginosa* [5]. The association of new β-lactamase inhibitors with FEP adds significant potential to the therapeutic armamentarium, increasing the spectrum of action and accelerating the scientific debate over sequential use of beta-lactams, carbapenem-sparing strategies, and overall Amp-C effects on vulnerable bacteria [5].

In recent years, new FEP combinations were introduced in daily clinical practice in infectious diseases such as cefepime/enmetazobactam (FEP/ENM), and some are near global approval such as cefepime/taniborbactam (FEP/TAN) and cefepime/zidebactam (FEP/ZID), or available in the future such as cefepime/nacubactam (FEP/NAC). This upgrade of cefepime that merged a well-known molecule with new β-lactamase inhibitors began a new era for this fourth-generation cefalosporin and has revitalized the role of cefepime in contemporary antimicrobial therapy.

In this review, we provide microbiological, pharmacological, and clinical data on the association of FEP with new β-lactam/β-lactamase inhibitors (BL/BLIs), and we foresee the potential use of new cefepime combinations in the current treatment strategies.

## 2. Materials and Methods

The current narrative review followed the Scale for the Assessment of Narrative Review Articles (SANRA) flow-chart. The main aim of this work is to summarize current evidence on new cefepime combinations.

A search was run on Google Scholar and PubMed using the terms (“Cefepime”) AND (“Enmetazobactam” OR “Zidebactam” OR “Taniborbactam” OR “Nacubactam”). Results were limited to papers published between 1 January 2000 and 1 April 2026. Studies were filtered for practice guidelines, guidelines, meta-analyses, systematic reviews, narrative reviews, case series, and case reports. The results were filtered, including only human patients over 18 years old.

Our search strategy permitted the identification of 98 papers, of which 30 were excluded by title and abstract evaluation. Then, the reviewers studied titles and abstracts. Subsequently, 68 papers were included. Finally, quality assessment of full-text studies was performed by two independent reviewers (VF and TL). Researchers reviewed the summary of all articles and ultimately used data from full articles to compile this review paper. Researchers assessed the inclusion of all titles and abstracts without language limitations in English. We duplicated other studies that previously included and excluded papers with no methods described, along with papers not strictly related to the aim of the study. The articles were classified according to journal importance and the number of references.

Variables were described with medians, absolute values, and rates.

## 3. New Cefepime Combinations

### 3.1. Cefepime/Enmetazobactam

ENM is a novel penicillanic acid sulfone-based BLI with potent inhibitory activity toward CTX-M, TEM, SHV, and other class A beta-lactamases [6]. ENM and tazobactam (TAZ) structures differ by the presence of a strategically placed methyl group, with overall maintained net neutral charge (zwitterion) that enhances its potency against Gram-negatives [6]. FEP/ENM was approved in 2024 by the FDA and EMA for the treatment of cUTIs including pyelonephritis, hospital-acquired pneumonia or ventilator-associated pneumonia (HAP/VAP), and bacteremia associated with cUTI or HAP/VAP [7,8], as reported in Table 1.

#### 3.1.1. In Vitro Activity

For antimicrobial susceptibility-testing purpose, the concentration of ENM is fixed at 8 µ g/mL and the effect of reduction in FEP MICs was investigated on 1696 clinical isolates of *Enterobacterales* from United States and Europe during 2014 and 2015, lowering FEP MIC90 from 16 to 0.12 µg/mL for *E. coli*, from >64 to 0.5 µg/mL for *K. pneumoniae*, from 16 to 1 µg/mL for *E. cloacae*, and from 0.5 to 0.25 µg/mL for *E. aerogenes*. For the subset of ESBL-producing microorganisms, the FEP MIC90 was reduced from >64 to 0.12 µg/mL and >64 to 1 µg/mL for *E. coli* and *K. pneumoniae* isolates, respectively [9].

ENM was shown to be more potent than TAZ against ESBL-producing isolates of K. pneumoniae, with MIC90 reduction higher than TAZ. Against *E. cloacae*, MIC90 values were lower than either Piperacillin/TAZ (PTZ/TAZ) or Ceftolozane/TAZ (CEF/TAZ) and comparable to Ceftazidime/Avibactam (CAZ/AVI) [9].

In a collection of 2627 *Enterobacterales* isolates from European hospitals during 2019–2021, the susceptibility was 97.9%, similar to Meropenem (MER) (97.3%) and CAZ/AVI (99.4%), higher than PTZ/TAZ (80.8%) and FEP alone (83.9%). Among the different species, susceptibility ranged from 93.9% (*K. pneumoniae*) to 100% (*E. coli*, *K. aerogenes*, *S. marcescens*, *P. mirabilis*, and *P. stuartii*), and 96.3% and 98.7% of *C. freundii* and *E. cloacae* isolates [10]. In the setting of those 320 isolates resistant to third-generation cephalosporins (3CEF-R) but susceptible to MER, with the majority (96.3%) represented by ESBL (mostly CTX-M-1), with or without Amp-C, the susceptibility was 99.1%, providing strong in vitro evidence of its potential clinical use against ESBL and Amp-C. ENM does not enhance the activity of FEP against *P. aeruginosa* [11].

From a molecular point of view, the diazabicyclooctane properties are consistent with the observation that ENM was not saturable with increasing concentrations of the inhibitor. Also, the methyl group on the triazole ring of ENM allows for many classical and nonclassical hydrogen bonding interactions (not observed for TAZ) in the active site of CTX-M-15 [6].

Resistance mechanisms include β-lactamases not inhibited by ENM and non-enzymatic mechanisms, such as modification of PBPs by target alteration, overexpression of efflux pumps, and outer membrane porin mutations, as reported in Table 2.

For *Enterobacterales*, EUCAST and CLSI set the susceptibility (clinical) breakpoint at ≤4 μg/mL and ≤8 μg/mL, respectively. For *P. aeruginosa*, only CLSI set the susceptibility breakpoint at ≤8 μg/mL [12].

#### 3.1.2. Preclinical In Vivo Data

Preclinical in vivo studies have consistently demonstrated that enmetazobactam successfully restores cefepime’s efficacy against multidrug-resistant Gram-negative pathogens. In a murine septicemia (peritonitis) model, the FEP/ENM combination showed robust therapeutic efficacy when mice were infected with challenging isolates, such as CTX-M-15/SHV-12/DHA-1 *K. pneumoniae*, CTX-M-15/OXA-48 *K. pneumoniae*, and CTX-M-15/AmpC *E. cloacae* [13]. These strains were highly resistant to cefepime alone due to the co-expression of multiple β-lactamases, but they were effectively neutralized by the FEP/ENM combination, demonstrating enmetazobactam’s ability to bolster cefepime’s therapeutic efficacy in vivo [13].

Furthermore, the in vivo pharmacodynamics of simulated human dosage regimens (such as FEP/ENM 2 g/0.5 g q8h) were evaluated in a neutropenic murine thigh infection model against a panel of 20 cefepime-nonsusceptible *Enterobacterales* strains [14]. While cefepime monotherapy failed, resulting in bacterial growth similar to untreated controls for 17 of the 20 strains, the humanized FEP/ENM regimen successfully induced a reduction of ≥0.5 log_10_ CFU for 12 of the 20 strains [14]. Increases in bacterial density were generally limited only to specific strains exhibiting highly elevated FEP/ENM MICs (>64 μg/mL), further confirming the strong predictive value of the drug’s in vitro potency [15].

The combination has also been rigorously tested in murine pneumonia models [16]. In severe pulmonary infections caused by ESBL-producing *K. pneumoniae*, the addition of enmetazobactam to cefepime facilitated significant bioburden reductions in the lungs [16]. Studies demonstrated a ≥2-log_10_ kill in the lung, establishing a strong preclinical foundation for its clinical approval and use in hospital-acquired and ventilator-associated pneumonia (HAP/VAP) [16,17].

#### 3.1.3. PK/PD Parameters

The PK profile of ENM (30 mg/kg) mirrored that of FEP (60 mg/kg) in a mouse septicemia model, and the addition of ENM did not alter the pharmacokinetics of FEP [8,16]. In a population PK analysis, there was no significant difference in FEP and ENM Cmax and AUC 0–24 between healthy volunteers and patients with cUTI. FEP is 20% bound to human plasma proteins, and the volume of distribution (Vd) at steady state is 20.02 L; plasma protein binding of ENM is negligible, and the mean Vd at steady state is 25.26 L. Mean clearance (CL) was 5.8 L/h for FEP, and 7.6 L/h for ENM, with similar terminal half-life (t1/2) (2.7 h and 2.6 h for FEP and ENM, respectively). For both FEP and ENM, unchanged renal excretion is the major elimination route [16,17].

Following administration of FEP/ENM 2/0.5 g q8h in patients with cUTI and eGFR ≥ 60 mL/min, the mean maximum concentrations (Cmax) at steady state (7 days) were 99.8 µg/mL and 19.8 µg/mL, respectively, while the mean plasma AUC 24 h time curve from time of dosing to the last measurable concentration (AUClast) were 379.5 µg·h/mL and 75.3 µg·h/mL, respectively [16,17].

In a neutropenic murine pneumonia model with ESBL-producing K. pneumoniae [16], it was concluded that the fT > threshold and fAUC:MIC were relevant PD indices; the AUC_ELF_/AUC_plasma_ were 73.4% and 61.5%, respectively, and a ≥2-log kill in the lung was achieved with a plasma FEP fT > MIC of ≥20% and ENM fT > 2 mg/liter of ≥20% of the dosing interval. The same targets have been subsequently used in the Phase I study on healthy volunteers [17].

The PK/PD profile of FEP/ENM in the human lung (pulmonary) environment [17] has been confirmed in 20 healthy volunteers receiving 2/1 g FEP/ENM iv q8h, with population pharmacokinetic modeling and Monte Carlo simulations: the mean (SD) percentage of partitioning of total drug concentrations between plasma and epithelial lining fluid (ELF) (AUCplasma/AUC_ELF_) were 60.59% (28.62%) and 53.03% (21.05%), respectively. At the dosage of 2/0.5 g IV q8h over a 2 h infusion, the probability of target attainment was ≥90% for *Enterobacterales* with a FEP/ENM MIC ≤ 8 mg/L [17].

#### 3.1.4. Clinical Data

The clinical efficacy and regulatory approval of FEP/ENM for complicated UTI (cUTI) and acute pyelonephritis (AP) are primarily anchored by the ALLIUM trial, a global, Phase III, randomized, double-blind, active-controlled, and multicenter study [18]. In this trial, hospitalized adults were randomized to receive intravenous FEP/ENM (2 g/0.5 g q8h, administered as a 2 h infusion) or piperacillin/tazobactam (PTZ 4 g/0.5 g q8h) for 7 days, with an extension up to 14 days in cases with concurrent bacteremia [18].

The primary endpoint was a composite of clinical cure (resolution of baseline signs and symptoms) and microbiological eradication (reduction in the baseline pathogen to <10^3^ CFU/mL in urine) assessed at the test-of-cure visit (day 14). Among the 1034 randomized patients, FEP/ENM successfully met both non-inferiority and superiority criteria versus PTZ, achieving the primary composite outcome in 79.1% of patients compared to 58.9% in the PTZ arm (treatment difference of 21.2%; 95% CI 14.3–27.9) [18]. This substantial benefit was predominantly driven by a significantly higher microbiological eradication rate (82.9% vs. 64.9%), while clinical cure rates remained comparably high between both groups (92.5% vs. 88.9%) [18].

Notably, FEP/ENM demonstrated pronounced benefits in high-risk patient subgroups, including the elderly, patients with moderate renal impairment, individuals with non-removable infection sources, high comorbidity burdens (Charlson Comorbidity Index > 3), and diabetes mellitus. A critical finding of the ALLIUM trial was the clear superiority of FEP/ENM in infections caused by ESBL-producing *Enterobacterales* (73.7% vs. 51.5%), as well as in patients presenting with bacteremia (71.1% vs. 50.0%) [18]. Furthermore, microbiological recurrence at day 14 was markedly lower with FEP/ENM compared to PTZ (11.3% vs. 29.4%). The safety profile of FEP/ENM was highly favorable and comparable to PTZ, with treatment-emergent adverse events occurring at similar frequencies (50.0% vs. 44.0%) and very low discontinuation rates due to side effects (1.7% vs. 1.5%) [18].

Regarding the treatment of hospital-acquired and ventilator-associated pneumonia (HAP/VAP), the EMA and FDA approvals of FEP/ENM were strategically based on a well-established PK/PD bridging approach rather than a dedicated Phase III clinical trial for pneumonia. This indication is supported by a Phase I intrapulmonary penetration study [17], which demonstrated that FEP/ENM achieves sufficient and proportional concentrations in the epithelial lining fluid (ELF). The high probability of target attainment in the lung tissue against target pathogens, combined with the proven clinical efficacy and safety established in the ALLIUM trial, provided the necessary regulatory foundation to extend its clinical use to severe respiratory tract infections [17,18].

#### 3.1.5. Dosage

The approved dosage is 2/0.5 gr q8h, 2 h infusion, which should be adjusted with renal impairment (eGFR < 60 mL/min) or augmented renal clearance (ARC) (≥130 mL/min); these recommendations are based on a single-dose study (NCT03680352) [19]. The respective dose fractions removed by hemodialysis were 0.30 and 0.35. In patients with ARC, prolongation of the infusion to 4 h is recommended [19].

### 3.2. Cefepime/Taniborbactam

Taniborbactam (TAN) is a highly innovative bicyclic boronic β-lactamase inhibitor [20,21]. Unlike traditional inhibitors, TAN exerts a potent inhibitory effect on both serine β-lactamases and metallo-β-lactamases (MBLs) [20,21]. This broad mechanism effectively restores the bactericidal activity of cefepime against various multidrug-resistant (MDR) and difficult-to-treat resistant (DTR) Gram-negative bacteria, including cefepime-non-susceptible *Enterobacterales*, *P. aeruginosa* expressing serine β-lactamases (such as KPC and OXA-48), carbapenem-resistant *P. aeruginosa* (CRPA), and *P. aeruginosa* with inducible AmpC production [20,21], as reported in Table 1 and Table 2.

#### 3.2.1. In Vitro Activity

TAN exhibits potent inhibition against *Enterobacterales* and *P. aeruginosa*, including isolates resistant to meropenem–vaborbactam, ceftazidime–avibactam, and ceftolozane–tazobactam [22,23]. The addition of TAN significantly reduces FEP MIC90 values by >64-fold against *Enterobacterales* and by 4-fold against *P. aeruginosa* [22,23]. Interestingly, TAN can restore cephalosporin activity against engineered CAZ-resistant *E. coli* strains expressing KPC variants by circumventing distinct effects on the hydrolytic activity and inhibition of KPC-2 versus KPC-3 [23]. Furthermore, FEP/TAN was active against *Enterobacterales* clinical strains carrying blaOXA-48, which presented a 78% rate of ESBL co-expression [24]. Regarding MBLs, FEP/TAN was highly active toward NDM-1 variants but remained poorly active against VIM-1-like enzymes [25,26,27,28].

#### 3.2.2. Preclinical In Vivo Data

In neutropenic murine cUTI and thigh infection models, using dosing regimens that simulate the human dose of 2 g/0.5 g q8h, the combination markedly enhanced the in vivo efficacy of FEP against *Enterobacterales* and *P. aeruginosa* isolates expressing a broad range of serine β-lactamases [21,22]. Specific considerations were drawn from a phase I trial in which 20 subjects underwent bronchoalveolar lavage (BAL) at different time points to measure drug concentrations in plasma, epithelial lining fluid (ELF), and alveolar macrophages (AM) [29]. The ELF-to-plasma AUC ratio was 0.17, while the Cmax for AM concentrations occurred at the last sampling time point (8 h post-administration), remaining constant over the dosing interval [29].

#### 3.2.3. PK/PD Parameters

In murine models, the optimal PK/PD indices were determined to be 50% fT > MIC for FEP and fAUC24/MIC for TAN [10,21,22]. In healthy human subjects, a multiple-dose scheme resulted in an AUC of 139.5 h·ng/mL, a volume of distribution (Vd) of 37.4 L, a half-life (t1/2) of 4.7 h, and a renal clearance of 5.6 L/h [30]. The mean fraction of the drug excreted unchanged in urine is 92.4% [30]. Significant and comparable reductions are predicted in drug clearance as renal function declines: the geometric mean clearance decreased by 18%, 63%, and 78% for FEP, and by 15%, 63%, and 81% for TAN in subjects with mild, moderate, and severe renal impairment, respectively [31]. Both drugs were found to be dialyzable, with comparable removal rates during a 4 h hemodialysis session [31].

#### 3.2.4. Clinical Data

The CERTAIN-1 phase III trial is a randomized, double-blind study in which hospitalized adults with cUTIs, including acute pyelonephritis (AP), were assigned in a 2:1 ratio to receive intravenous (IV) FEP/TAN 2.5 g or meropenem 1 g q8h for 7 days (with a possible extension to 14 days in cases of concurrent bacteremia) [32]. Favorable results were observed in ESBL-producing infections, and superiority was achieved in the composite outcome of clinical and microbiological efficacy, demonstrating higher frequencies of success at late follow-up in isolates susceptible to both compared drugs [32]. The safety profile was similar for both treatments [32]. However, the CERTAIN-2 study, which compared the combination against meropenem in hospitalized adult patients with pneumonia, was withdrawn [33]. Ultimately, the FDA rejected the approval for cUTI in February 2024 [34].

#### 3.2.5. Dosage

Although TAN is currently not approved by either the FDA or the EMA, the anticipated dosage matches that of FEP/ENM (2 g/0.5 g q8h), based on preliminary PK/PD data [31,33] and the CERTAIN-1 study [32]. Dose variations according to renal function are expected [33,34]. Currently, no data are available regarding the optimal dosage in patients with severe renal impairment undergoing hemodialysis or continuous veno-venous hemofiltration (CVVH) [31,33].

### 3.3. Cefepime/Zidebactam

Zidebactam (ZID) is a second important new diazabicyclooctane (DBO) compound [35,36]. It functions as a non-β-lactam inhibitor of β-lactamases, possessing a wide spectrum of activity against all Ambler classes (A–D) when used in combination with FEP [35,36]. Notably, this molecule, similar to nacubactam (NAC), retains direct intrinsic antibacterial activity by binding to PBP2, which creates a powerful enhancer effect alongside PBP3-targeting β-lactams against *Enterobacterales* [35,36], as reported in Table 2.

#### 3.3.1. In Vitro Activity

Extensive data from a large cohort (N = 7876) of clinical isolates are available detailing the in vitro activity of FEP/ZID against *Enterobacterales* (N = 5946), *P. aeruginosa* (N = 1291), and *Acinetobacter* spp. (N = 639) sourced from the United States, Europe, Asia-Pacific, and Latin America [37]. Overall, there is strong homogeneity in susceptibility among *Enterobacterales* and *P. aeruginosa*, despite lower activity against *Acinetobacter* spp. [37]. A subsequent analysis by the same group confirmed its activity against various clinically relevant β-lactamases, including ESBLs, KPCs, AmpC, and MBLs [37]. Two important additional considerations must be noted. First, FEP/ZID demonstrated poor activity against *P. aeruginosa* and *A. baumannii* strains exhibiting resistance or reduced susceptibility to cefiderocol [38,39]. Second, although FEP/ZID shows strong activity against diverse, carbapenem-non-susceptible, and colistin-resistant *K. pneumoniae* isolates producing OXA-48-like and/or NDM carbapenemases, preliminary findings of FEP/ZID resistance among carbapenemase-producing *E. coli* suggest potential PBP2 target mutations [38,39].

#### 3.3.2. Preclinical In Vivo Data

The robust activity of FEP/ZID was well demonstrated in murine pneumonia models against carbapenem-resistant *A. baumannii* (CRAB), CRPA, and carbapenem-resistant *Enterobacterales* (CRE) [40,41,42,43,44,45,46]. Bhagwat et al. demonstrated that achieving a 1-log_10_ bacterial kill required cefepime to maintain concentrations above the MIC for 38.9% of the dosing interval [45]. However, when combined with a non-effective dose of ZID, this requirement significantly decreased to 15.5% [47]. In the murine thigh model, FEP/ZID showed equivalent efficacy against CRAB and CRPA (meropenem MICs ranging from 8 to >256 μg/mL) [45]. Monogue et al. reported strong in vivo activity of the FEP/ZID combination against all tested isolates, with ZID alone showing enhanced efficacy in 11 out of 16 isolates (mean bacterial reduction with FEP/ZID: −1.62 ± 0.58 log_10_ CFU/thigh) [43].

#### 3.3.3. PK/PD Parameters

Phase 1 trials (NCT02674347, NCT02707107) revealed that ZID and FEP/ZID are safe and well-tolerated in healthy adults, displaying linear pharmacokinetics and dose-proportional increases in Cmax and AUC24 [48,49]. Rodvold et al. evaluated ELF and AM concentrations of FEP/ZID (2 g/1 g q8h), demonstrating that the respective AUC24 values based on mean ELF concentrations were 127.9 mg·h/L for FEP and 52.0 mg·h/L for ZID, and 87.9 mg·h/L for FEP and 13.2 mg·h/L for ZID in AM [48]. These findings robustly support further studies in pneumonia [50]. In a single-center PK evaluation of FEP/ZID in subjects with renal impairment, Preston et al. observed a t1/2 of 2.56 h and a clearance (CL) of 102 mL/min in healthy normal subjects, compared to a t1/2 of 10.4 h and a CL of 25.7 mL/min in subjects with severe renal impairment (mean creatinine clearance of 17.98 mL/min) [49].

#### 3.3.4. Clinical Data

No results are currently available from the phase III, randomized, double-blind, multicenter, comparative study (NCT04979806) evaluating hospitalized adults with cUTIs and AP treated with either FEP/ZID (2 g/1 g q8h) or meropenem (1 g q8h) [50]. However, there are successful case reports detailing the compassionate use of FEP/ZID for DTR *P. aeruginosa* and CRPA strains [51,52,53].

#### 3.3.5. Dosage

No optimal dosage has yet been definitively established, although phase I and PK/PD studies suggest a regimen of FEP/ZID 3 g (2 g/1 g) q8h [53,54]. Considering that ZID is predominantly excreted through the kidneys, dose adjustments or reductions will likely be required in patients with mild-to-severe renal impairment [53,54].

### 3.4. Cefepime/Nacubactam

Nacubactam (NAC), formerly known as OP0595, is another novel DBO non-β-lactam inhibitor of β-lactamases that provides a wide spectrum of activity against all Ambler classes (A–D) when combined with FEP [55,56,57,58]. NAC also possesses other intriguing properties, including intrinsic PBP2-targeted antibacterial activity against Enterobacterales and the ability to act as an enhancer for β-lactam agents that primarily target PBP3 [56], as reported in Table 1 and Table 2.

#### 3.4.1. In Vitro Activity

Extensive data exist regarding NAC’s activity against MBL producers, encompassing *Citrobacter* spp., *Enterobacter* spp., *E. coli*, *Klebsiella* spp., and *Proteeae* (i.e., *Morganella* spp. and *Providencia* spp.) strains [59,60]. Combinations such as FEP/NAC and meropenem/NAC demonstrate broad activity against MBL producers, regardless of the specific MBL type [59,60]. However, this activity varies between species, with higher FEP/NAC MICs observed more frequently among *Proteeae* [59,60].

#### 3.4.2. Preclinical In Vivo Data

Data from two phase I in vivo studies utilizing a neutropenic murine pneumonia model are available [61]. The results showcased excellent in vivo activity against CRE *E. cloacae* and ESBL-producing *K. pneumoniae* in the mouse models [61]. In another phase I preclinical in vivo study employing a neutropenic murine thigh infection model, the MICs of FEP/NAC (evaluated using a 1:1 ratio) were 2- to >128-fold lower than those of FEP alone against the studied CRE *K. pneumoniae* and *E. coli* strains, reinforcing that the most critical PK/PD index for β-lactams remains the %fT > MIC [62].

#### 3.4.3. PK/PD Parameters

Two phase I studies evaluated the PK and safety of single and multiple escalating doses of NAC in healthy volunteers [61]. The pharmacokinetics of NAC following single (1000 to 8000 mg) and multiple (1000 to 4000 mg) doses were linear, and primary PK parameters were unaffected by the infusion duration [61]. The majority of the administered NAC doses were excreted unchanged via the kidneys, with a minor contribution from non-renal elimination processes (estimated renal clearance ranged from 5.5 to 8.5 L/h) [61]. NAC penetration into the ELF was evaluated in a non-randomized, open-label study in healthy volunteers completed in 2018 (NCT03182504), but no data are currently available [62]. Furthermore, no data on the PK/PD parameters of the FEP/NAC combination in healthy volunteers have been published yet [63]. However, an estimated 30% fT > MIC target was reported for monitoring the FEP/NAC combination to achieve a static effect against β-lactamase-producing *Enterobacterales* [63].

#### 3.4.4. Clinical Data

Two multicenter, randomized, phase III clinical studies are currently underway [64,65]. The Integral-1 study (NCT05887908) was designed to assess the safety and efficacy of FEP/NAC (2 g/1 g q8h) or aztreonam/NAC (2 g/1 g q8h) compared to imipenem/cilastatin (1 g/1 g q8h) in the treatment of cUTIs and HAP [64]. Although this trial has been completed, no results are currently available [64]. The Integral-2 study (NCT05905055) is actively recruiting to evaluate the safety and efficacy of FEP/NAC and aztreonam/NAC compared to the best available therapy in adults with infections caused by CRE isolates, including cUTIs, AP, HAP, VAP, and cIAIs [65].

#### 3.4.5. Dosage

No optimal dosage has been established yet, although phase I and PK/PD studies seem to suggest FEP-NAC 3 g (2 g/1 g) q8h. Dose adjustment with reduced renal function may be foreseen.

### 3.5. The Evolution of Cefepime: FEP 2.0 Strategies

The revitalization of FEP through its combination with novel BLIs—such as ENM, TAN, ZID, and NAC—is set to significantly expand the therapeutic armamentarium against MDR infections. This evolution necessitates careful consideration from microbiological, pharmacological, and clinical perspectives. Beyond simple efficacy, the integration of these BL/BLI combinations into antimicrobial stewardship (AMS) protocols is complex; it involves managing secondary resistances and implementing sequential ᵝ-lactam strategies to preserve drug utility.

#### 3.5.1. Microbiological and Clinical Efficacy

**Figure 1:**
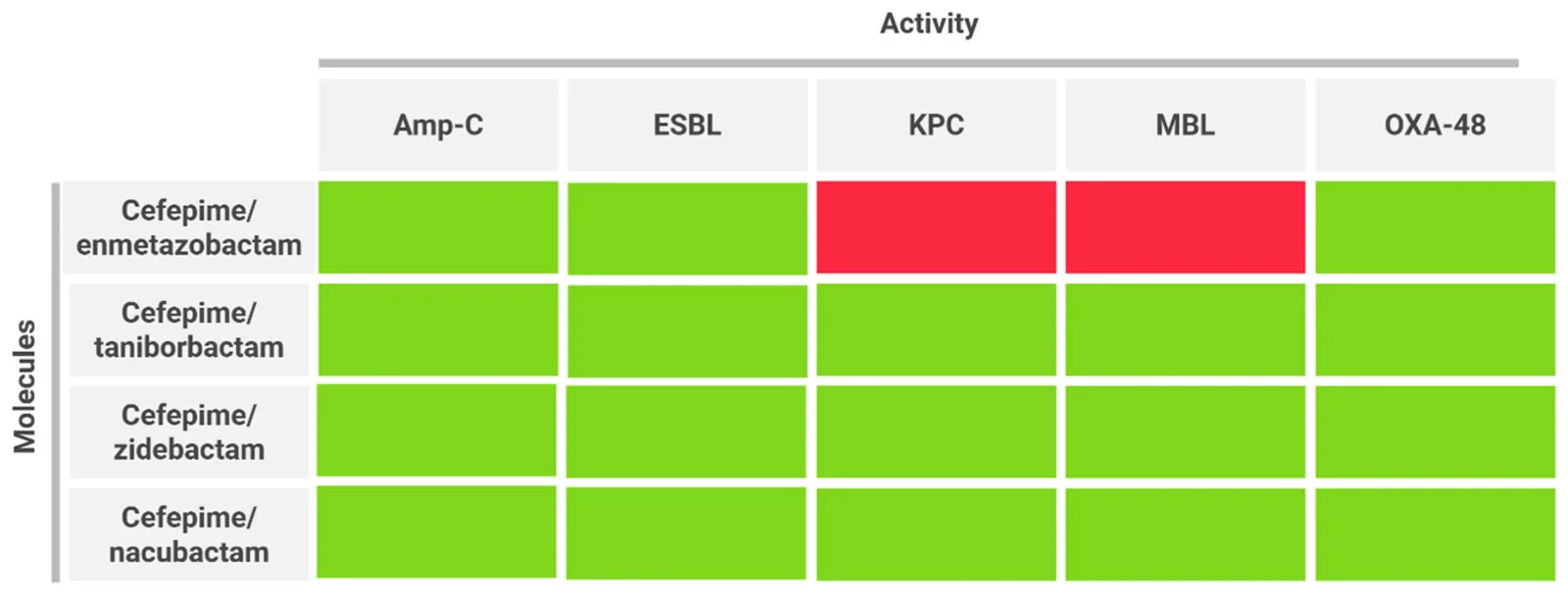
Antimicrobial spectrum of new cefepime combinations against most frequent resistance mechanisms.

From a microbiological standpoint, each novel BLI possesses distinct structural and mechanistic properties, broadening FEP’s spectrum to include Amp-C, OXA-48, ESBLs, KPC, and MBLs, as reported in Figure 1.

While FEP combined with TAN, ZID, or NAC demonstrates activity against carbapenem-resistant strains, FEP-ENM is primarily limited to Amp-C, ESBL-producing pathogens, and OXA-48-producing strains. However, this should not be viewed as a deficiency. Rather, it represents a highly effective carbapenem-sparing tool. The ALLIUM trial confirmed this by demonstrating a significantly higher microbiological cure rate for FEP-ENM compared to MER in patients with cUTIs, establishing it as a viable alternative for Amp-C or ESBL-producing infections [18]. Historically, such carbapenem-sparing roles were attributed to CAZ-AVI and CEF/TAZ, which, however, should be reserved for KPC-producing *Enterobacterales* or DTR- P. aeruginosa infections, respectively. Today, FEP-based regimens offer an attractive alternative to carbapenems, potentially mitigating the selection pressure that drives carbapenemase-producing epidemics. The traditional “carbapenem–ESBL–carbapenem” cycle has long been a driver of resistance, particularly as carbapenems are potent inducers of AmpC enzymes.

#### 3.5.2. Clinical Application and Empirical Use: Will “FEP 2.0” Strategies Render Older Agents Obsolete?

Likely not. Despite the caveats of the MERINO trial, PTZ/TAZ remains a valid carbapenem-sparing option for empiric treatment in non-severe, non-immunocompromised patients [66,67]. Nevertheless, FEP-ENM is increasingly preferred for cUTI patients with risk factors for ESBLs (e.g., prior hospitalization or cephalosporin exposure) or, given its high microbiological activity combined with low microbiological recurrence rate, for those at high risk of relapse, such as the elderly and immunosuppressed.

**Figure 2:**
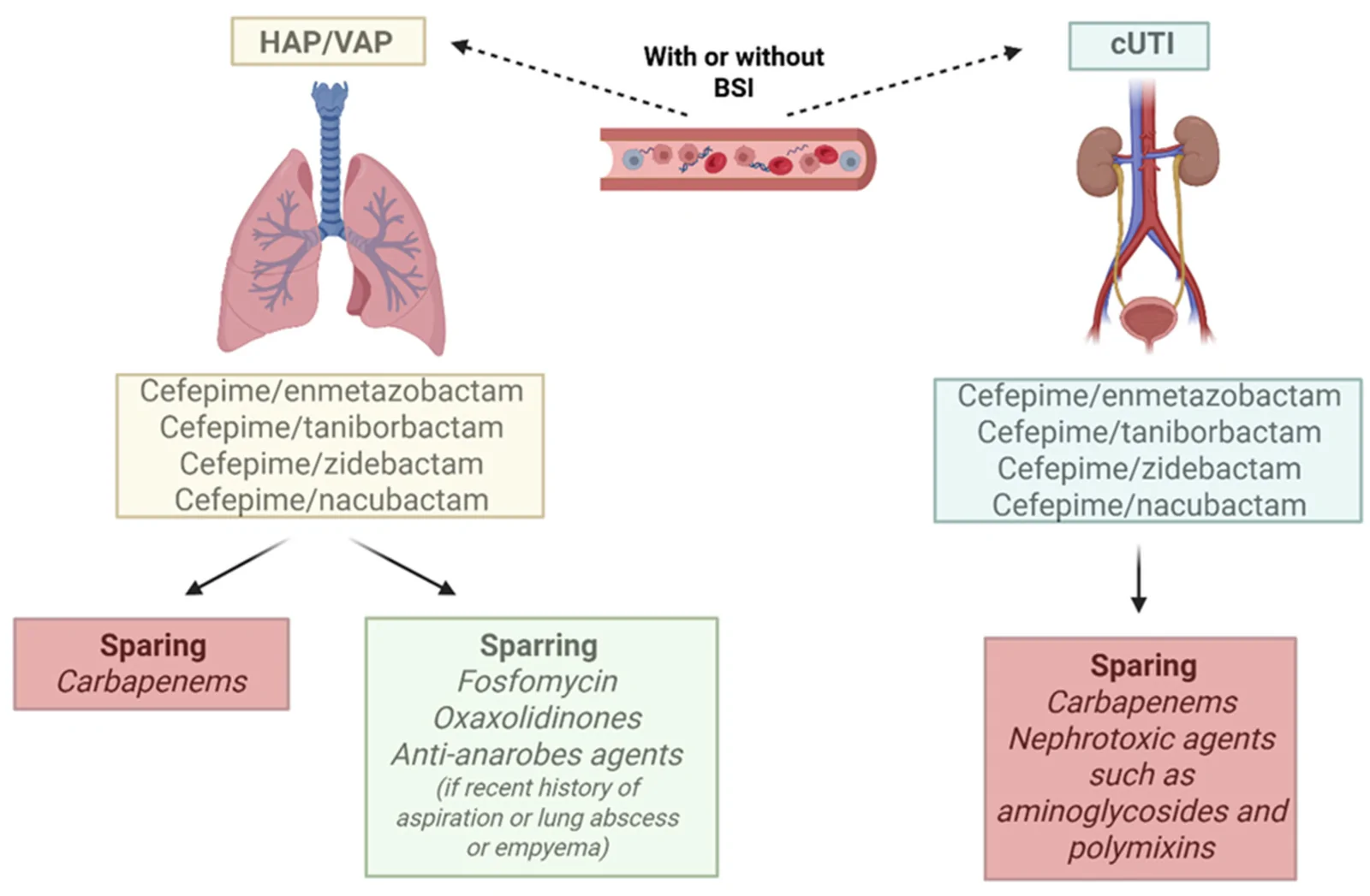
Graphical stewardship approach in HAP/VAP and cUTIs of the new FEP combination in patients at risk for MDR pathogens.

While CAZ-AVI and CEF/TAZ remain cornerstones for KPC/OXA-48 and P. aeruginosa infections [68,69,70,71], rising resistance rates (up to 10% in some KPC strains) and the epidemiological shift toward MBLs necessitate the adoption of FEP-TAN, FEP-ZID, and FEP-NAC. The activity of these combinations against MBLs provides a critical advantage, especially as current options are limited to complex combinations like CAZ + AZT or the use of cefiderocol. In Figure 2, we described a graphical stewardship approach in HAP/VAP and cUTIs of the new FEP combination in patients at risk for MDR pathogens.

Looking ahead, FEP 2.0 strategies show great promise for treating nosocomial pneumonia. Adequate penetration into the lung ELF is vital for efficacy and to prevent resistance. FEP alone demonstrates a mean bronchial mucosa penetration of approximately 59.8%. When paired with ENM, both agents exhibit linear pharmacokinetics and similar partitioning into the ELF (for FEP and for ENM), suggesting robust performance in HAP/VAP scenarios [16,17].

**Figure 3:**
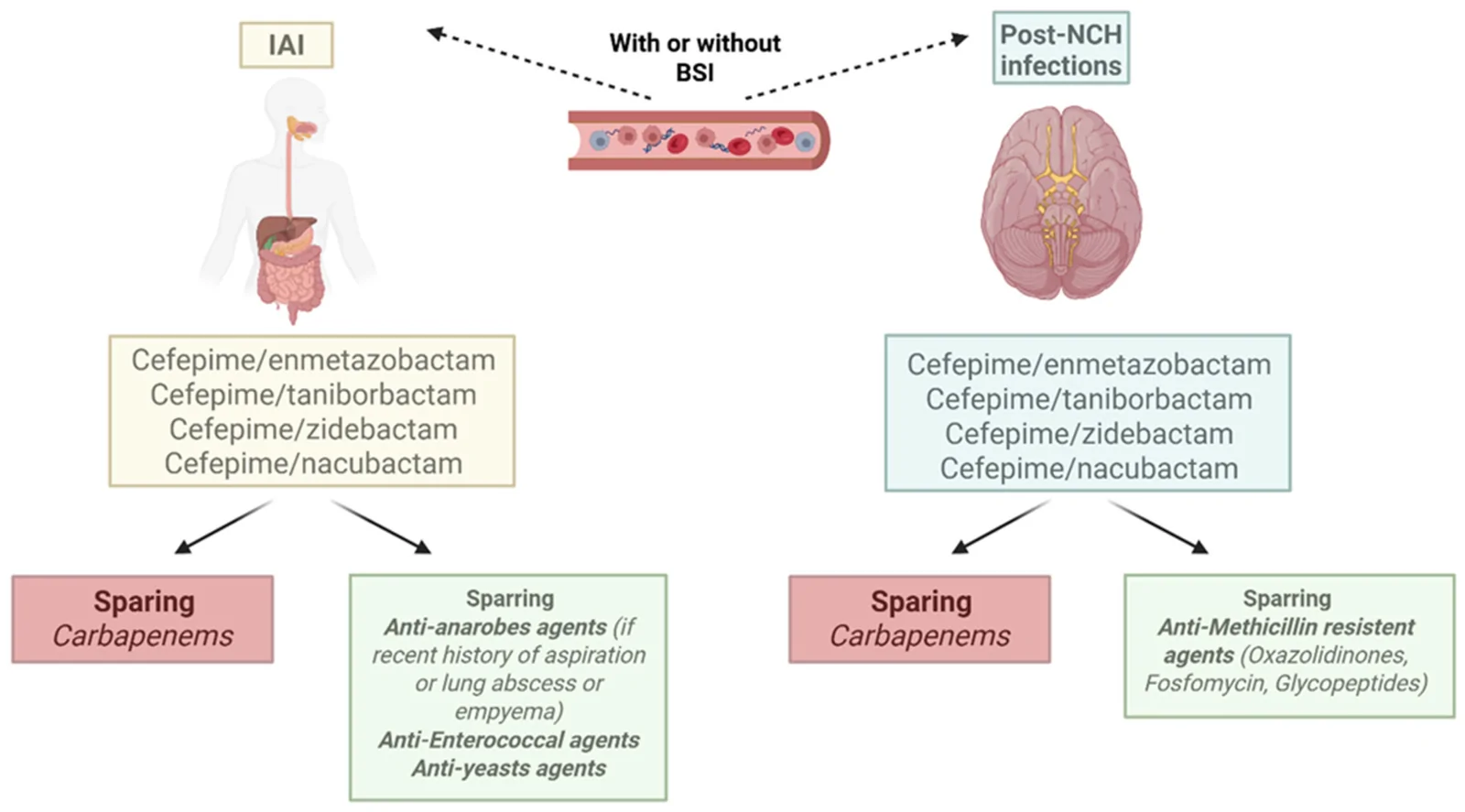
Unmet clinical need in which FEP new combinations could be useful.

Moreover, despite the lack of PK/PD data, randomized trials and real-world evidence, barring some case reports, new FEP combinations in the future could cover the gap in treatment in specific settings in which MDR pathogens with a high variability between centers have raised particular attention in recent years, such as in IAI and post-neurosurgical infections, as reported in Figure 3.

However, certain limitations must be acknowledged. FEP 2.0 combinations lack anaerobic coverage and offer limited activity against Gram-positive pathogens, including methicillin-resistant *S. aureus* and *Enterococcus* spp. Furthermore, the risk of *Clostridioides difficile* must be monitored; in the ALLIUM trial [18], a slightly higher incidence was noted in the FEP-ENM cohort compared to PTZ/TAZ.

The paramount importance of this review lies in providing a structured, evidence-based framework to navigate the newly expanded “Cefepime 2.0” landscape, bridging the gap between emerging pharmacological data and daily clinical practice. As the therapeutic armamentarium against multidrug-resistant Gram-negative pathogens grows, these novel β-lactamase inhibitor combinations cannot be viewed as uniform or interchangeable assets. Accordingly, based on our findings and the proposed stewardship algorithms, we outline specific clinical recommendations. First, as illustrated in Figure 1 and Figure 2, clinicians should proactively position FEP/ENM as a frontline carbapenem-sparing agent for complicated urinary tract infections (cUTIs) and hospital-acquired/ventilator-associated pneumonia (HAP/VAP) driven by ESBL-, AmpC-, and OXA-48-producing pathogens. This targeted use not only limits the evolutionary pressure exerted by carbapenems but also spares the use of highly nephrotoxic agents like aminoglycosides and polymyxins. Second, FEP/TAN, FEP/ZID, and FEP/NAC should be strictly preserved as targeted rescue therapies for more challenging carbapenem-resistant isolates, specifically KPC- and MBL-producing pathogens (Figure 1), where current therapeutic options are severely limited. Finally, we recommend extending the evaluation of these combinations to critical unmet clinical needs, such as post-neurosurgical infections and complicated intra-abdominal infections (Figure 3), provided that concurrent anti-anaerobic or Gram-positive coverage is added when clinically indicated. Ultimately, the successful deployment of these FEP 2.0 combinations mandates a precision-medicine approach, requiring clinicians to integrate local epidemiological data, specific resistance profiles, and optimal PK/PD exposures within robust antimicrobial stewardship programs.

## 4. Conclusions

The emergence of novel cefepime-based β-lactam/β-lactamase inhibitor combinations marks a pivotal evolution in the management of difficult-to-treat Gram-negative infections. “FEP 2.0” strategies redefine the positioning of cefepime within modern antimicrobial stewardship frameworks, bridging the gap between carbapenem-sparing approaches and the need for effective therapies against increasingly complex and frequently co-expression of different resistance mechanisms, including ESBLs, AmpC, KPC, OXA-48, and MBL-producing pathogens.

Importantly, the distinct microbiological spectra of ENM, TAN, ZID, and NAC should not be interpreted as interchangeable assets, but rather as complementary tools requiring tailored clinical application according to local epidemiology, source of infection, patient severity, and prior antimicrobial exposure. In this context, FEP-ENM may emerge as a valuable carbapenem-sparing strategy for ESBL- and AmpC-producing *Enterobacterales*, whereas FEP-TAN, FEP-ZID, and FEP-NAC could play an increasingly relevant role against CRE, including MBL, particularly as resistance to currently available agents continues to rise.

Beyond microbiological efficacy, the future success of FEP 2.0 strategies will depend on their integration into precision stewardship models aimed at optimizing early adequate therapy: sequential β-lactam strategies, sparing and sparing strategies, PK/PD optimization, and careful patient selection need to be the central components of clinical evaluation.

In conclusion, cefepime is no longer merely an established fourth-generation cephalosporin, but the backbone of a new generation of adaptable therapeutic platforms. Whether FEP 2.0 strategies will significantly improve the management of MDR infections and reduce carbapenem usage will depend on the ability of clinicians, microbiologists, and stewardship teams to optimize the use of those new drugs in a continuously evolving epidemiological landscape.

## Figures and Tables

**Table 1:** Main information regarding new cefepime combinations, approval, indications, and drug dosing.

Drug Combination	Primary Trials & Registry	Study Phase	Comparators	Indications	Key Outcomes/Findings	Dose of Drug	Safety and Adverse Events
Cefepime/enmetazobactam (FEP/ENM)	ALLIUM; NCT03680612; NCT03680352	Phase III (ALLIUM); Phase II (NCT03680612)	Meropenem (ALLIUM); Cefepime alone (Phase II)	cUTIs including pyelonephritis, HAP/VAP, and bacteremia associated with cUTI or HAP/VAP	FDA and EMA approved in 2024. Demonstrated superiority over meropenem for cUTI. Positioned as a carbapenem-sparing tool.	2/0.5 g q8h administered as a 2 h infusion	Safety profile highly favorable and comparable to PTZ. Low discontinuation rates. A slightly higher incidence of *C. difficile* infection was noted compared to PTZ.
Cefepime/taniborbactam (FEP/TAN)	CERTAIN-1; CERTAIN-2 (Withdrawn); NCT06168734	Phase III	Meropenem (1 g q8h)	cUTI, acute pyelonephritis (AP), and VABP/Ventilated HABP	Superiority achieved in CERTAIN-1 for cUTI. However, FDA rejected approval for cUTI in February 2024. Restores activity against MBLs (NDM-1 variants).	2.5 g (2/0.5 g) q8h	Safety and tolerability profile similar to meropenem
Cefepime/nacubactam (FEP/NAC)	Integral-1 (NCT05887908); Integral-2 (NCT05905055)	Phase III	Imipenem-cilastatin (Integral-1); Best Available Therapy (Integral-2)	cUTI/AP; infections due to CRE (HABP, VABP, cIAI)	Integral-1 is completed, but results are pending. Integral-2 is currently recruiting. Broad activity against MBL producers.	3 g (2 g/1 g) q8h	Safe and well-tolerated based on Phase I healthy volunteer studies. Phase III safety data are currently pending

**Table 2:** Cefepime combinations and key microbiological features.

Cefepime Combination	Resistance Mechanisms Covered (Ambler Classes)	Spectrum and Key Microbiological Features
**Cefepime/enmetazobactam (FEP/ENM)**	Class A (ESBLs like CTX-M, TEM, SHV) Class C (AmpC) Also active against OXA-48 (Class D; *preclinical in vivo data*) *Not active on class B (MBLs)*	Primarily targets ESBL-producing Enterobacterales. It does not enhance cefepime’s activity against *P. aeruginosa*. Potent carbapenem-sparing tool for susceptible infections.
**Cefepime/taniborbactam (FEP/TAN)**	Class A (including KPC) Class B (MBLs like NDM-1) Class C (AmpC) Class D (OXA-48)	Restores activity against carbapenem-resistant *P. aeruginosa* (CRPA) and Enterobacterales. While active against NDM-1 variants, it shows poor activity against VIM-1-like metallo-β-lactamases.
**Cefepime/zidebactam (FEP/ZID)**	All Ambler classes (A–D), including ESBLs, KPCs, AmpC, and MBLs (NDM and OXA-48-like).	High activity against Enterobacterales and *P. aeruginosa.* Zidebactam acts as a β-lactam enhancer by binding to PBP2, which complements cefepime’s PBP3 targeting. It has lower activity against *Acinetobacter* spp.
**Cefepime/nacubactam (FEP/NAC)**	All Ambler classes (A–D), including ESBLs, AmpC, KPC, OXA-48, and MBLs.	Demonstrates wide activity against MBL producers regardless of the specific MBL type. Like zidebactam, it has intrinsic antibacterial activity by targeting PBP2 in Enterobacterales. Activity varies by species, with higher MICs noted in *Proteeae*.

## Data Availability

No new data were created or analyzed in this study. Data sharing is not applicable to this article.

## Abbreviations

The following abbreviations are used in this manuscript:

AMR	Antimicrobial resistance
FEP	Cefepime
cUTIs	Complicated urinary tract infections
SSTIs	Skin and soft tissue infections
cIAIs	Complicated Intra-Abdominal infections
PBPs	Penicillin-binding proteins
BL	Beta-lactams
BLIs	Beta-lactamase inhibitors
ENM	Enmetazobactam
TAZ	Tazobactam
HAP	Hospital-acquired pneumonia
VAP	Ventilator-associated pneumonia
CEF	Ceftolozane
CAZ	Ceftazidime
AVI	Avibactam
PTZ	Piperacillin
MER	Meropenem
3CEF-R	Resistant to third-generation cephalosporins
ELF	Epithelial lining fluid
Vd	Volume of distribution
CL	Mean clearance
Cmax	Mean maximum concentration
ARC	Augmented renal clearance
TAN	Taniborbactam
MBLs	Metallo-beta-lactamases
MDR	Multidrug resistant
DTR	Difficult-to-treat
CRPA	Carbapenem-resistant *P. aeruginosa*
MEV	Meropenem–vaborbactam
AM	Alveolar macrophages
CVVH	Continuous veno-venous hemofiltration
ZID	Zidebactam
CRAB	Carbapenem-resistant *A. baumannii*
NAC	Nacubactam
CRE	Carbapenem-resistant *Enterobacterales*
CPE	Carbapenem-producing *Enterobacterales*
AZT	Aztreonam
AMS	Antimicrobial stewardship
